# Entropy of the Universe and Hierarchical Dark Matter

**DOI:** 10.3390/e24081171

**Published:** 2022-08-22

**Authors:** Paul H. Frampton

**Affiliations:** Dipartimento di Matematica e Fisica “Ennio De Giorgi”, Università del Salento and INFN-Lecce, Via Arnesano, 73100 Lecce, Italy; paul.h.frampton@gmail.com

**Keywords:** entropy, holographic bound, dark matter, primordial black holes

## Abstract

We discuss the relationship between dark matter and the entropy of the universe, with the premise that dark matter exists in the form of primordial black holes (PBHs) in a hierarchy of mass tiers. The lightest tier includes all PBHs with masses below one hundred solar masses. The second-lightest tier comprises intermediate-mass PIMBHs within galaxies, including the Milky Way. Supermassive black holes at galactic centres are in the third tier. We are led to speculate that there exists a fourth tier of extremely massive PBHs, more massive than entire galaxies. We discuss future observations by the Rubin Observatory and the James Webb Space Telescope.

## 1. Introduction

In particle theory, the concept of entropy is generally not fundamental, because for one elementary particle entropy is neither defined nor useful.

In general relativity and cosmology, the situation is different. For black holes, entropy is a central and useful concept. In cosmology, the entropy of the universe has often been considered, although not sufficiently emphasised. We shall argue that the origin and nature of cosmological dark matter can be best understood by consideration of the entropy of the universe. We made such an argument four years ago [1], but that discussion was too diluted by considering simultaneously the dark matter being made from elementary particles such as WIMPs and axions, as favoured three decades ago [2].

In this paper, we dispose of microscopic candidates in one paragraph. The standard model of particle theory (SM) has two examples of lack of naturalness: the Higgs boson and the strong CP problem. Our position is that to understand these, we still need to better understand the SM itself. Regarding the strong CP problem, it is too ad hoc to posit a spontaneously broken global symmetry and consequences which include an axion. Concerning the WIMP, the idea that dark matter experiences weak interactions arose from assuming TeV-scale supersymmetry, which is now disfavoured by the LHC data. To identify the dark matter, we instead look up.

Assuming dark matter is astrophysical and that the reason for its existence lies in the second law of thermodynamics, we shall be led uniquely to the dark matter constituents being primordial black holes (PBHs). We must admit that there is no observational evidence for any PBHs, but according to our discussion PBHs must exist. In the ensuing discussion, we shall speculate that they exist in abundance in four tiers of mass, up to and including several galactic masses.

Because PBH entropy is related to mass squared, we are mainly interested in masses satisfying $M_{PBH}>100M_{⊙}$. From here on, we shall adopt the unadorned acronym PBH to denote only those with masses which satisfy $M<100M_{⊙}$. In the earliest discussions of PBHs, they were tacitly assumed to be this light, even usually much lighter than the Sun. This lightest tier will contribute a negligible fraction of the total dark matter entropy but can contribute a few percent to the total dark matter mass. Within the Milky Way, we use the acronym PIMBH for intermediate-mass PBHs in the mass range $10^{2}M_{⊙}<M_{PIMBH}<10^{5}M_{⊙}$. Outside the Milky Way, we entertain all masses $10^{2}M_{⊙}<M_{PBH}<10^{17}M_{⊙}$. Of these, we use the term PSMBH for supermassive PBHs in the mass range $10^{5}M_{⊙}<M_{PSMBH}<10^{11}M_{⊙}$ and PEMBH for extra-massive PBHs with $10^{11}M_{⊙}<M_{PEMBH}<10^{17}M_{⊙}$.

Although the visible universe (VU) is not a black hole, its Schwarzschild radius is about 68% of its physical radius, i.e., 30 Gly versus 44 Gly, so it is close. This curious fact seems to have no bearing on the nature of the dark matter. A few more acronyms will be useful: CMB, CIB and CXB. CMB is the familiar cosmic microwave background, while I and X refer to infrared and X-ray, respectively.

There exist a number of constraints on PBHs derived from astronomical observations [3,4]. We would advise caution in interpreting constraints derived from CMB distortion caused by additional microwaves resulting from X-ray emission by accreted matter. The accretion model often used is of a spherically symmetric Bondi type, which can overestimate accretion by as much as four orders of magnitude and hence lead to constraints which are far too stringent. This is not to say that all such constraints are wrong, only that they do not follow from the arguments given.

The arrangement of the paper is as follows. We discuss entropy and the second law in Section 2 and Section 3, and then in Section 4, we discuss primordial black holes. In Section 5 and Section 6, we discuss two methods of PBH detection: microlensing and the cosmic infrared background, respectively. Finally, in Section 7, we discuss our results.

## 2. Entropy

We begin with the premise that the early universe can be regarded in an approximate sense as a thermodynamically isolated system, for the purposes of our discussion. It certainly contains a number of particles, ∼$10^{80}$, vastly larger than the numbers normally appearing in statistical mechanics, such as Avogadro’s number, ∼$6\times 10^{23}$ molecules per mole.

No heat ever enters or leaves, and it can be considered as though its surface were covered by a perfect thermal insulator. It is impracticable to solve all the Boltzmann transport equations, so it is mandatory to use thermodynamic arguments, provided that we may argue that the system is proximate to thermal equilibrium.

In 1872, making the then-unsupported assumption [5] of atoms and molecules, Boltzmann discovered the quantity S(t) in terms of the molecular momentum distribution function f(p,t)

(1) S(t)=−∫dpf(p,t)logf(p.t)

which satisfies

(2) $$\left(\frac{dS(t)}{dt}\right)\geq 0$$

and can be identified with the thermodynamic entropy. The crucial inequality, Equation (2), the second law, was derived in [5] for non-equilibrium systems, assuming only the Boltzmann transport equations and the ergodic hypothesis.

Ascertaining the nature of dark matter can be regarded as a detective’s mission, and there are useful clues in the visible universe. In [1], we made an inventory of the entropies of the known objects in the visible universe using a venerable source, i.e., Weinberg’s book [6]. Let us model the visible universe as containing $10^{11}$ galaxies, each of mass $10^{12}M_{⊙}$ and each containing one central SMBH with mass $10^{7}M_{⊙}$. We recall that the dimensionless entropy of a black hole is $S/k\left(M_{BH}=\eta M_{⊙}\right)∼10^{78}\eta^{2}$. Then, the inventory gives:

- SMBHs ∼$10^{103}$;
- Photons ∼$10^{88}$;
- Neutrinos ∼$10^{88}$;
- Baryons ∼$10^{80}$.

We regard this entropy inventory as a **first clue**. From the point of view of entropy, the Universe would be only infinitesimally changed if everything except the SMBHs were removed. This suggests more generally that black holes totally dominate the entropy, as we shall find in the following.

A second remarkable fact about the visible universe is the near-perfect black-body spectrum of the CMB, which originated some 300,000 years after the beginning of the present expansion era, or after the Big Bang in more familiar language. We are not tied to a Big Bang, which could well be replaced by a bounce in a cyclic cosmology.

The precise CMB spectrum is a **second clue** about dark matter. It suggests that the plasma of electrons and protons prior to recombination is in excellent thermal equilibrium, and hence the matter sector was in thermal equilibrium for the first 300,000 years. This, combined with the thermal isolation mentioned already, underwrites the use of entropy and the second law during this period.

A **third clue**, the final one about dark matter, lies in the holographic principle [7], which provides, as an upper limit on the entropy of the visible universe, the area of its surface in units of the Planck length. Given its present co-moving radius of 44 Gly, this requires $S/k\leq 10^{123}$. The entropy of the contents which is so bounded might nevertheless tend to approach [8] a limit that is many orders of magnitude higher than the total entropy in the limited inventory listed above.

## 3. Second Law

For primordial black holes (PBHs) formed at cosmic time *t*, their mass may be taken to be governed by the horizon size, giving

(3) $$M_{PBH}=10^{5}M_{⊙}\left(\frac{t}{1\sec}\right)$$

so that PBHs with masses $10^{2}M_{⊙}<M_{PBH}<10^{17}M_{⊙}$ are produced for $10^{-3}$ s < *t* < 30 ky. The top few orders of magnitude are unlikely, but possible.

A tendency to increase the entropy of the universe towards $S_{U}/k$∼$10^{123}$ can be most readily achieved by the formation of PBHs, the more massive the better because $S_{BH}/k$∼$10^{78}\eta^{2}$ for mass $M_{BH}=\eta M_{⊙}.$ For example, in the case where a PEMBH existed with $M_{PEMBH}$∼$10^{17}M_{⊙}$, it would have S/k∼$10^{112}$, which is a billion times the entropy of the items listed in our previous inventory.

The PBH mass function is unknown, so we must make reasonable conjectures which may approximate Nature. For a preliminary discussion, we may take monochromatic distributions separately for PIMBHs, PSMBHs and PEMBHs. The real mass function is expected to be smoother, but the general features in our discussion of entropy should remain valid.

In a toy model for the visible universe, we include $10^{11}$ galaxies each with mass $10^{12}M_{⊙}$. As hierarchical dark matter, we shall take as an illustration all PIMBHs with $100M_{⊙}$, all PSMBHs with $10^{7}M_{⊙}$ and all PEMBHs with $10^{14}M_{⊙}$. Let the number of each type be $n_{I}$, $n_{S}$ and $n_{E}$, respectively. The total dark matter mass is then

(4) $$M=\left(10^{2}n_{I}+10^{7}n_{S}+10^{14}n_{E}\right)M_{⊙}$$

while the total entropy contributed by all PBHs is

(5) $$S/k=\left(10^{82}n_{I}+10^{92}n_{S}+10^{106}n_{E}\right)$$

Let us begin with the middle of the three hierarchical tiers, the supermassive black holes known to reside in galactic centres. In our toy model, $n_{S}$ is equal to the number of galaxies, $n_{S}=10^{11}$, so their total mass and entropy are, from Equation (4),

(6) $$M\left(PSMBHs\right)=10^{18}M_{⊙}$$

and from Equation (5),

(7) $$S\left(PSMBHs\right)/k=10^{103}$$

Before considering Equations (4) and (5) further, let us step back and ask which of the three terms in each equation is most likely to be dominant. The answer is different for Equations (4) and (5), because entropy S/k and mass *M* have the relationship $S/k\propto M^{2}$.

The total mass in Equation (4) is comparable to the total mass of the visible universe, which is ∼$10^{123}M_{⊙}$. Comparison with M(PSMBHs) in Equation (6) then shows that the second term on the right-hand side of Equation (4) is sub-dominant, being several orders of magnitude less than the left-hand side.

Now, let us discuss the first term on the right-hand side. In our toy model, every galaxy has mass $10^{12}M_{⊙}$, dominated by the dark matter halo made up of $100M_{⊙}$ PIMBHs, and therefore, since there are $10^{11}$ galaxies, we take $n_{I}=\left(10^{11}\right)\times \left(10^{10}\right)=10^{21}$, whereupon the total mass and entropy of the PIMBHs are, from Equation (4),

(8) $$M\left(PIMBHs\right)=10^{23}M_{⊙}$$

and from Equation (5),

(9) $$S\left(PIMBHs\right)/k=10^{103}$$

From Equation (8), we deduce that the first term on the right-hand side of Equation (4) is a dominant term. We already know that the second term on the right-hand side is relatively small. What about the third and last term? At this stage, we can say little, except that observation is consistent with it vanishing. Perhaps surprisingly, to jump ahead, after discussion of the entropy equation, Equation (5), we shall suggest that the third term on the right-hand side of Equation (4) is comparable to the first term on the right-hand side of Equation (4), thus providing a rather novel viewpoint for dark matter.

Substituting our choices $n_{I}=10^{21}$ and $n_{S}=10^{11}$ into Equation (5), we find the total entropy

(10) $$S/k=\left(2\times 10^{103}+10^{106}n_{E}\right)$$

to be compared to the total mass

(11) $$M=\left(10^{23}+10^{18}+10^{14}n_{E}\right)M_{⊙}$$

In Equation (11), for consistency, we must bound the parameter $n_{E}$ from above by $n_{E}\leq 10^{9}$, to avoid overclosing the universe. It is interesting to study the upper limit of $n_{E}$ in the entropy equation, Equation (10). This gives ∼$10^{115}$, to be compared with the holographic bound on the entropy [7,8], which is ∼$10^{123}$.

In the absence of any observational evidence about either dark matter or primordial black holes, we must look at the visible universe from the two theoretical viewpoints of mass and entropy. This suggests the most likely scenario, which is $n_{E}$∼$10^{9}$. This predicts that our toy universe contains of the order of one billion extra-massive black holes with masses $O(10^{14}M_{⊙})$, or perhaps a smaller number of even more massive PBHs. Because of their extraordinarily high masses, these PEMBHs are not expected to be associated with a specific galaxy or cluster of galaxies.

## 4. Primordial Black Holes

If black holes make up all the dark matter, they cannot be all gravity-collapse black holes, due to baryon-number conservation. The amount of dark matter is more than five times that of baryons. Therefore, most or all dark matter black holes must instead be primordial.

PBHs are black holes formed in the early universe when there was high density and sufficiently large fluctuations and inhomogeneities. Their existence was first conjectured in the 1960s in the Soviet Union [9] and independently in the 1970s in the West [10]. Initially, it was realised that only PBHs with mass greater than $10^{-18}M_{⊙}$ could survive to the present time, due to Hawking evaporation. Nevertheless, it was generally assumed that PBHs were all very much lighter than the Sun and hence even lighter than all the PBHs considered in the bulk of this paper.

During this early era of extremely light PBHs, the seminal idea that PBHs could form all the dark matter was proposed in 1975 by Chapline [11]. In 2009 [12] and in 2010 [13], the relevance of entropy in cosmological evolution emerged.

Beginning in 2010 [14], the upper limit on PBH mass was removed by showing that in a specific model of hybrid inflation, with two stages of inflation, a parametric resonance could mathematically yield fluctuations and inhomogeneities of arbitrarily large size. We regard this as merely an existence theorem; such formation might take place without inflation.

The possibility of PBHs with many solar masses led to the 2015 dark matter proposal in [15] that PIMBHs are an excellent astrophysical candidate for dark matter in the Milky Way halo, especially given the absence of a compelling elementary particle candidate, either within the standard model or in any plausible extension thereof. This was further underscored in [16]. Both these papers emphasised microlensing by PIMBHs of starlight from the Magellanic Clouds [17] as a promising method for the detection of PIMBHs in the Milky Way.

These PIMBHs are now to be regarded as the second of four mass tiers, the third being the supermassive PSMBHs at galactic centres and the fourth being extremely massive PEMBHs, more massive than galaxies. The first tier contains all PBHs with masses below $100M_{⊙}$.

Returning to our thermodynamic arguments about entropy, we use the entropy inventory of the known entities to propose the idea that very massive black holes already dominate the entropy through the PSMBHs, which we assume are primordial because there seems to be insufficient cosmic time for stellar-mass black holes to grow adequately by accretion and mergers.

For the entropy of the universe to be nearer to its holographic upper limit, we are led to introduce $10^{9}$ PEMBHs of $10^{14}M_{⊙}$, in order to reach S/k∼$10^{115}$. Achieving the maximum S/k∼$10^{123}$ is possible with just ten PEMBHs of $10^{22}M_{⊙}$. If true, this would be revolutionary.

We do not expect PEMBHs to be associated, in general, with specific luminous galaxies or clusters of galaxies, so we do not discuss here the interesting topic of co-evolution [18]. Nevertheless, it is interesting to learn that for masses >$10^{12}M_{⊙}$, accretion should proceed [19] in a non-luminous manner, so that such a PEMBH can never appear in a quasar.

A Kerr black hole is characterised by only three parameters, M,S and *Q*, and in astrophysics it was common to assume that the electric charge vanishes. Recent papers [20,21,22,23] have seriously queried this assumption for PSMBHs. For example, in [20], an upper limit on a non-zero electric charge of the Milky Way’s PSMBH, $SgrA^{*}$, has been given as $3\times 10^{8}$ C. The exciting possibility of non-vanishing electric charges for PEMBHs also merits sedulous study.

PEMBHs have also been discussed by Carr et al. in [24]. We have already mentioned that one of the first proposals of PBHs involved Carr in 1974 [10]. Nobody has contributed more papers on the study of PBHs than Carr, as exemplified by papers in 1975 [25], 2010 [26] and 2016 [27].

## 5. Microlensing

Gravitational lensing of a distant star by a nearer massive object or lens moving across the field of view gives rise to an enhancement of the star and to a temporal light curve whose duration is proportional to the square root of the mass of the lens, as displayed in Equation (12).

As already mentioned, a direct way to discover PIMBHs in the Milky Way would be to use microlensing [15,16] of the light from the stars in the Magellanic Clouds. Assuming a transit velocity of 200 km/s, an estimate of the duration $\hat{t}$ of the light curve at half maximum is given by

(12) $$\hat{t}∼0.2y{\left(\frac{M_{lens}}{M_{⊙}}\right)}^{\frac{1}{2}}$$

which means that for $10^{2}M_{⊙}<M_{PIMBH}<10^{5}M_{⊙}$, the duration of the light curve is in the range $2y<\hat{t}<60y$. Masses below $2500M_{⊙}$ with $\hat{t}<10y$ are clearly the most practicable to measure.

A successful precursor was an experiment by the MACHO Collaboration [17] in the 1990s. In the 2020s, microlensing searches at the Vera Rubin Observatory [28] were able to repeat this success for the much higher mass ranges of the MACHOs expected for the dark matter inside the Milky Way.

The MACHO Collaboration, 1992–1999, used the observatory at Mount Stromlo near Canberra, Australia. This was a 1.27 m telescope with two 16-megapixel cameras. The project showed that the technique could be used successfully to discover MACHOs, as well as confirming this prediction of Einstein’s general relativity theory. The highest duration of their more-than-a-dozen light curves was 230 days, corresponding to a mass close to $10M_{⊙}$.

An attempt was made to use the Blanco 4m telescope at Cerro Tololo, Chile with the 570-megapixel DECam, in order to find light curves with durations of two years or more, and hence, by Equation (12), lenses with $M>100M_{⊙}$. The longer durations led, however, to crowding in the field of view such that it was impracticable to track a specific target star.

A more powerful telescope under construction at Cerro Panchon, also in Chile, is the Vera Rubin Observatory telescope [28], expected to start taking data in 2023. This is an 8.4 m telescope with a 3.2-gigapixel camera, both significantly larger than previously, and we can reasonably hope that it can microlens multi-year-duration light curves and possibly confirm the existence of PIMBHs in the Milky Way.

## 6. Cosmic Infrared Background

At large redshifts Z>15, a population of PBHs would be expected to accrete matter and emit X-ray and UV radiation which will be redshifted into the CIB, to be probed for the first time by the James Webb Space Telescope [29], which could therefore provide support for PBH formation.

Analysis of a specific PBH formation model [30] supports the idea that the JWST observations in the infrared could provide relevant information about whether PBHs really were formed in the early universe.

This is important because, although we have plenty of evidence for the existence of black holes, whether any of them are primordial is not known. The gravitational wave detectors [31] LIGO, VIRGO and KAGRA have discovered mergers in black hole binaries with initial black holes in the mass range 3–85 $M_{⊙}$. We suspect that all or most of these are not primordial, but that is only conjecture.

The supermassive black holes at galactic centres, including Sgr A* at the centre of the Milky Way, are well established and are primordial in our toy model. Whether that is the case in Nature is unknown.

Because of the no-hair theorem that black holes are completely characterised by their mass, spin and electric charge (usually taken to be zero), there is no way to tell directly whether a given black hole is primordial or the result of the gravitational collapse of a star.

The distinction between a primordial and a non-primordial black hole can be made only from knowledge of its history. For example, if it existed before star formation, it must be primordial. The infrared data from JWST might be able to provide useful insight into this central question.

## 7. Discussion

It is familiar to study a mass–energy pie-chart of the universe with approximately 5% baryonic normal matter, 25% dark matter and 70% dark energy. The entropy pie-chart is very different if the toy model considered in this paper resembles Nature. The slices corresponding to normal matter and dark energy are extremely thin, and the pie is essentially all dark matter.

In this article, we have attempted to better justify the discussion in our previous 2018 paper [1], which argued that entropy and the second law applied to the early universe provide a *raison d’être* for the dark matter. In [15,16], we proposed that the dark matter constituents in the Milky Way are PIMBHs, a second tier of PBH beyond the light ones with masses of less than one hundred solar masses.

Here, we have included the supermassive PSMBHs at the galactic centres as a third tier of dark matter with a similar primordial origin, to replace the conventional wisdom that SMBHs arise from accretion and merging of black holes which arise from the gravity collapse of stars.

We have gone one step further and discussed a fourth tier of extremely massive PEMBHs, more massive than clusters, whose entropy far exceeds that of PIMBHs and PSMBHs. If this is correct, then although normal matter contributes as much as 5% of the mass–energy pie-chart of the universe, its contribution to an entropy pie-chart is truly infinitesimal.

Since it has never been observed except by its gravity, it does seem most likely that dark matter has no direct or even indirect connection to the standard model of strong and electroweak interactions in particle theory, including the extensions thereof aimed at ameliorating problems with naturalness existing therein with respect to the Higgs boson and the strong CP problem.

The three clues we mentioned in the Introduction, i.e., the dominance of black holes in the entropy inventory, the CMB spectrum and the holographic entropy maximum all hint at PBHs as the dark matter constituents.

One ambiguity is whether the maximum entropy limit suggested by holography should be saturated, in which case the mass function for the PEMBHs must be extended to high values.
